# Using multiclass classification to automate the identification of patient safety incident reports by type and severity

**DOI:** 10.1186/s12911-017-0483-8

**Published:** 2017-06-12

**Authors:** Ying Wang, Enrico Coiera, William Runciman, Farah Magrabi

**Affiliations:** 1 Centre for Health Informatics, Australian Institute of Health Innovation, Macquarie University, Sydney, 2109 NSW Australia; 2 Centre for Population Health Research, Division of Health Sciences, University of South Australia, Adelaide, Australia; 3 Australian Patient Safety Foundation, Adelaide, Australia

**Keywords:** Machine learning, Patient safety, Text mining, Incident reporting, Medical informatics

## Abstract

**Background:**

Approximately 10% of admissions to acute-care hospitals are associated with an adverse event. Analysis of incident reports helps to understand how and why incidents occur and can inform policy and practice for safer care. Unfortunately our capacity to monitor and respond to incident reports in a timely manner is limited by the sheer volumes of data collected. In this study, we aim to evaluate the feasibility of using multiclass classification to automate the identification of patient safety incidents in hospitals.

**Methods:**

Text based classifiers were applied to identify 10 incident types and 4 severity levels. Using the one-versus-one (OvsO) and one-versus-all (OvsA) ensemble strategies, we evaluated regularized logistic regression, linear support vector machine (SVM) and SVM with a radial-basis function (RBF) kernel. Classifiers were trained and tested with “balanced” datasets (n__*Type*_ = 2860, n__*SeverityLevel*_ = 1160) from a state-wide incident reporting system. Testing was also undertaken with imbalanced “stratified” datasets (n__*Type*_ = 6000, n__*SeverityLevel*_ =5950) from the state-wide system and an independent hospital reporting system. Classifier performance was evaluated using a confusion matrix, as well as F-score, precision and recall.

**Results:**

The most effective combination was a OvsO ensemble of binary SVM RBF classifiers with binary count feature extraction. For incident type, classifiers performed well on balanced and stratified datasets (F-score: 78.3, 73.9%), but were worse on independent datasets (68.5%). Reports about falls, medications, pressure injury, aggression and blood products were identified with high recall and precision. “Documentation” was the hardest type to identify. For severity level, F-score for severity assessment code (SAC) 1 (*extreme risk*) was 87.3 and 64% for SAC4 (*low risk*) on balanced data. With stratified data, high recall was achieved for SAC1 (82.8–84%) but precision was poor (6.8–11.2%). *High risk* incidents (SAC2) were confused with *medium risk* incidents (SAC3).

**Conclusions:**

Binary classifier ensembles appear to be a feasible method for identifying incidents by type and severity level. Automated identification should enable safety problems to be detected and addressed in a more timely manner. Multi-label classifiers may be necessary for reports that relate to more than one incident type.

**Electronic supplementary material:**

The online version of this article (doi:10.1186/s12911-017-0483-8) contains supplementary material, which is available to authorized users.

## Background

Approximately 10% of admissions to acute-care hospitals are associated with an adverse event (an incident resulting in patient harm) [1, 2]. An event or circumstance that could have resulted, or did result, in unnecessary harm to a patient is called a patient safety incident. The reporting of patient safety incidents is now widespread and is regarded as a cornerstone of initiatives to improve the safety of health services [3]. Incident reports are a critical resource for understanding how and why incidents occur. Analysis of narratives about adverse events and near misses can inform policy and practice for safer care. Timely analysis and response to the growing volume of reports about such patient safety incidents are urgent challenges.

With widespread use of centralized reporting systems, the volume of incident reports has increased. Unfortunately, our capacity to monitor and respond to these reports in a timely manner is limited by the sheer volumes of data collected. For instance, 492,526 incidents were reported to the UK National Reporting and Learning System from April to June 2015, a 15.8% increase from the previous year [4]. Current methods, which rely upon the retrospective manual review of reports, can no longer keep up with the growing volume of incidents being reported by healthcare workers [5–7].

The use of incident reports to examine a specific patient safety problem such as falls or medications, is highly dependent on identifying these *incident types* from the large volume of reports collected within an incident monitoring system database. *An incident type is a descriptive term for a category of incidents with a common nature, grouped because of shared, agreed features* [8]*.* Reported incidents can vary in severity and the likelihood of recurrence. Most reporting systems apply a *severity level* to grade the seriousness of an incident, to prioritise investigation of high-risk events. This is critical in ensuring that events with significant consequences which are likely to recur are immediately followed-up.

The efficiency of identifying incident type and severity can be improved by asking reporters to identify incident type and severity when they are first recorded. A major problem with this approach is that incidents are reported by healthcare workers from a range of professional groups including clinicians, hospital administrators and safety officers who may not be expert in incident classification [9–12]. Problems with asking reporters to identify incidents are well documented in the literature [13]. For example, in one controlled experiment which used video-based scenarios to examine the categorisation of falls by 446 staff from seven hospitals, there was no consensus about what constituted a fall in five out of the 14 scenarios tested [14]. Falls onto surfaces higher than the ground were less likely to be considered as incidents. The type of hospital and ward also influenced whether a scenario was categorised as a fall.

Similarly, a severity rating system only works if reporters are knowledgeable about the system, and are able to apply their knowledge consistently. Severity ratings assigned by healthcare workers are often inconsistent. A study carried out to evaluate the reliability of the severity rating scale used by the UK National Reporting and Learning System for medication errors showed that there are marked differences in the severity ratings between different health professional groups, within groups and for individuals at different time points [13], making severity rating highly subjective [15]. Further, reporters are often hesitant in assigning high severity ratings and many reports are submitted without a severity rating, missing opportunities for preventive and corrective actions. In the US, 25% of reports submitted to an incident reporting system were labeled as “miscellaneous” and “other” [16].

One way of improving the efficiency and accuracy of identifying incident reports is to automatically classify the incidents using text classification techniques. In healthcare, text classification has been used to identify adverse drug events in discharge summaries [17], surgical margin status in pathology reports [18] and disorders in chest radiograph reports [19]. Other studies have sought to identify reports about patient safety incidents using unsupervised methods [20–22]. In our preliminary work, we showed the feasibility of using supervised methods based on statistical text classification to identify reports about three types of incidents: patient identification [23], clinical handover [23], and health information technology [24]. We developed binary classifiers based on Naïve Bayes, logistic regression and Support Vector Machines (SVM) and subsequently showed that extreme-risk events could be identified using a similar approach [25]. Others have similarly showed the feasibility of text classification to identify reports involving health information technology [26]. However, these studies have focused on distinguishing a specific incident type from all other types.

In reality, safety improvement initiatives are interested in many different types of incidents and severity levels reflecting the range of safety problems encountered in healthcare. This extension from binary classification, addressed by our previous studies, to multiclass classification is not straightforward. The complexity of multiclass problems increases with the number of classes due to noise and variance among input variables which poses a challenge for well-known classification methods, especially with limited training data [27]. Binary classifiers are good at discriminating one class from another but do not perform as well when more classes are involved [28]. In this study, we thus set out to explore the real-world multiclass problem where reports need to be categorized into many different incident types and severity levels.

Our aim was to evaluate the feasibility of using multiclass classification to automate the identification of reports about safety problems in hospitals by type and level of severity. We focused on ten patient safety problems that are recognized as priority areas for safety and quality improvement (Table 1) [29–31]. One of the most popular techniques for addressing such multiclass classification problems is to reduce the problem into multiple binary classification problems [32], as the decision boundaries are simpler than when considering all classes within a single optimization formula. We sought to examine different combinations of binary classifier ensembles, feature extraction methods, and decision-making schemes. Classifiers were built separately for incident type and severity level using balanced datasets for training, validation and testing. We then examined generalizability by testing on imbalanced stratified datasets which represented real-world conditions.

**Table 1:** Text classifiers were trained to identify reports about 10 safety problems in hospitals by type and severity level. This table shows the composition of balanced and stratified datasets used for classifier training and testing

	balanced AIMS *benchmark*	stratified AIMS *original*		stratified Riskman *independent*	
	*n*	*n*	%	*n*	%
Incident type					
Falls	260	90	20	872	15
Medications	260	68	15	1053	18
Pressure injury	260	37	8	190	3
Aggression	260	49	11	487	8
Documentation	260	26	6	252	4
Blood product	260	5	1	59	1
Patient identification	260	7	2	86	1
Infection	260	6	1	22	<1
Clinical handover	260	7	2	87	1
Deteriorating patient	260	1	<1	14	<1
Others	260	148	33	2878	48
Total	2860	444		6000	
Severity level					
SAC1	290	25	<1	23	<1
SAC2	290	95	2	105	2
SAC3	290	2198	45	2609	44
SAC4	290	2519	52	3213	54
Total	1160	4837		5950	

## Methods

There are two classic ways to address multiclass classification: problem transformation and algorithm adaptation. In problem transformation, multiclass classification is converted into a series of binary classification sub-tasks, while algorithm adaptation handles multiclass data directly using a single optimization formula. Problem transformation is a natural choice for patient safety incident reports as it provides a simpler approach; binary classifiers to handle individual incident types are easier to implement and computationally efficient. In general, the complexity of multiclass classification increases with the number of classes. With limited training data, algorithm adaptation methods are harder to optimize while problem transformation is adaptive. For example, new classes can be easily incorporated by training additional binary classifiers with no changes to the original classifiers.

We decomposed our multiclass classification problem into a series of binary classification problems. Two traditional binary classifier ensemble strategies, one-versus-one (OvsO) and one-versus-all (OvsA), were used to combine base binary classifiers [32]. The OvsO strategy transforms an *l* class problem into l*(l-1)/2 binary problems by involving all possible combinations between pairs of classes, where base classifiers are responsible for distinguishing between pairs of classes. In prediction, new samples are presented to each binary classifier and their output is combined to give the predicted class. The OvsA strategy divides an *l* class problem into *l* binary problems by training classifiers to distinguish one class from all other classes. Prediction is based on the binary classifier with the highest output probability.

We chose two discriminative base binary classifiers, logistic regression model and support vector machines (SVM) rather than generative classifiers (e.g. Naïve Bayes) because discriminative classifiers have almost invariably outperformed others in similar text classification of high-dimensionality data with limited training samples. SVM-based classifiers are the state-of-the-art for many text classification tasks, despite the proposal of new approaches that work much better for other tasks, as they tend to generalize well when tested on independent data. Two SVM kernel types were considered, linear and radial-basis function (RBF). Kernel parameters (γ for an RBF kernel) and the trade-off parameter (C) were tuned to optimize classifiers.

### Identifying training and evaluation datasets

We used reports from two separate incident monitoring systems, the Advanced Incident Management System (AIMS) [33] and Riskman [34]. AIMS is based on 20 years of research in patient safety, and has been used since 1998 in many facilities in Australia, New Zealand, South Africa, and the United States. In Australia, it has been used across the public hospital system in four of the eight states and territories: New South Wales, Western Australia, South Australia, and the Northern Territory. These jurisdictions account for approximately 60% of the population of Australia and receive high numbers of incident reports per year. The Riskman system is an independent tool used across the state of Victoria and a number of private hospitals across the country.

For classifier training and testing, we used 6000 randomly selected reports from 137,522 submitted to AIMS across an Australian state between January and December 2011. To test classifier generalizability, an independent set of 6000 reports were randomly selected out of 28,159 submitted to Riskman in a teaching hospital between January 2005 and July 2012. Incident reports consist of a number of structured and free text fields used to describe the event and its consequences (see Additional file 1: Appendix A). The mean word length for the free text in reports was 78.5 in AIMS (range: 5–308, SD: 35.5) and 63.4 in Riskman (range: 5–404, SD: 31.6). The seriousness of an incident is graded using an internationally accepted rating system called the severity assessment codes (SAC). SAC was developed by the US Veterans Administration, and assignment of risk is based on the severity of an incident and the likelihood of recurrence [2]. One of four risk ratings (i/ extreme; ii/ high; iii/ medium; iv/ low) is assigned by reporters upon submission [35].

Upon collection, all reports in the training and test sets were read and any identifiable or potentially patient identifying information was removed in accordance with jurisdictional privacy requirements (e.g. name, date of birth). Three experts in the classification of patient safety incidents reviewed and validated the labels for the 10 incident types recognized as priority areas for safety and quality improvement (Additional file 1: Appendix B) [29–31]. These areas were chosen prior to the data collection. Inter-rater reliability for determining incident types was Cohen’s kappa = 0.93 (*p* < 0.001 95% CI 0.9301–0.9319). Using a random sampling approach a further set of unrelated incidents were also labeled to ensure representativeness of the ‘Others’ set (i.e. including ten other types, see Additional file 1: Appendix B). The labels provided by the experts were used as a “gold standard” for training and testing the performance of classifiers. For severity level, the gold standard was based on SAC ratings which were checked and ratified by local managers who had received training in assessing severity levels and were familiar with the nature of incidents and their consequences. Ethical approval was obtained from university committees as well as a committee governing the hospital and state datasets.

### Data preparation

Only descriptive narratives in reports were used for experiments including incident description, patient outcome, actions taken, prevention steps, investigation findings and results. All codes, punctuation and non-alphanumerical characters were removed and text was converted to lower case.

### Experimental setup

We used 260 samples for each incident type and 290 reports for each SAC level for classifier training (Table 1); these sample sizes were based on our previous studies [23, 25]. Balanced AIMS datasets were used for training. For testing, balanced AIMS datasets were firstly used to generate ***benchmark*** results. Classifiers were then applied to imbalanced “stratified” datasets from AIMS (***original***) and Riskman (***independent***) to evaluate their applicability in real-world conditions and to examine generalizability. The stratified datasets were constructed so that the distribution of incident types and severity levels was representative of their real-world ratio (Table 1).

### Experimental workflow

**Fig. 1:**
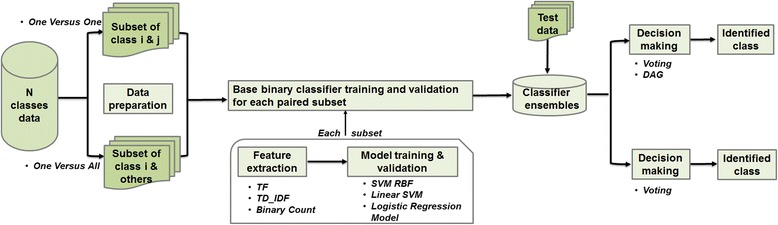
Experimental workflow to train and evaluate classifiers to identify reports by type and severity level (TF: term frequency; TD_IDF: term frequency-inverse document frequency, DAG: directed acyclic graph)

An overview of our approach is shown in Fig. 1. Experiments comprised four main tasks. First, datasets were decoded into two-class subsets according to OvsO or OvsA ensemble schemes. For the OvsA ensembles, samples from all other classes were randomly selected to create evenly distributed subsets of 260 or 290 reports. The narratives of reports were then processed into more informative representations via feature selection and extraction methods. Next, binary classifiers were trained and validated for pairs of classes using cross-validation. Then, two widely-used group decision-making schemes, voting and directed acyclic graph (DAG) [36], were used to identify incidents in testing sets by combining results from all base binary classifiers. Finally, performance was evaluated. Each of these four tasks is detailed below.

1. Feature extraction
  The goal of feature extraction is to transform the raw input data, such as text, into numerical representations interpretable by classifiers while providing discriminative information for classification. To enhance the quality of feature extraction, text pre-processing methods including removal of stop words and short words with fewer than two characters, stemming, and lemmatization were applied to the reports [37].
  We then adopted a bag-of-words model commonly used in document classification to extract features [38]. Irrespective of grammar, incident narratives were represented as an unordered collection of words, and unique words were used as features. The bag of words was then transformed into a numeric representation using three different feature extraction methods, binary count, term frequency (tf), term frequency-inverse document frequency (tf-idf). Binary count transforms individual processed reports into 1 or 0 corresponding word occurrences while Tf converts reports into the actual frequency of word occurrences. When a word appears in many reports, it is considered relatively common but less important. To evaluate the importance of words tf-idf was adopted, this transforms reports into the term frequency of each word multiplied by the inverse document frequency [39].
2. Base classifier training and validation
  To train the base classifiers, a 10-fold repeated random sub-sampling cross-validation method was used to assign incidents to training (80%), validation (10%), and testing (10%) sets. For each incident type, we randomly selected 10% of reports and set them aside for testing. This was done because we were concerned that, with random assignment, a testing report for one base binary classifier might be used in the training set of another base classifier. For example, a report i about a fall incident which was used for training a base classifier (versus medications), could be assigned to the test set of another base classifier (versus blood product). For training and validation, the folds were created using repeated random sub-sampling. Using this strategy classifiers were built and validated to avoid potential overlaps between training and testing sets.
  We did not give preference to any types when training base binary classifiers. The loss function was set to improve the F-score, which was the equally weighted harmonic mean of the precision and recall. For instance, the kernel size for SVM RBF classifier was optimized during validation to achieve the best F-scores. Classifiers that achieved higher accuracy were adopted in ensembles for testing.
3. Group decision-making schemes
  Voting is the most common group decision-making scheme, where each classifier votes and the final prediction is based on the class with the most votes [40]. This works for both OvsO and OvsA ensemble strategies, while DAG only applies to OvsO [32]. Starting at the root node, DAG makes a binary decision by rejecting either class. It then moves forward along the un-rejected branch to reach a leaf node that is the predicted class (Fig. 2). Compared with voting, DAG has the same number of training steps generating l*(l-1)/2 binary classifiers but only requires *l-1* comparisons.

**Fig. 2:**
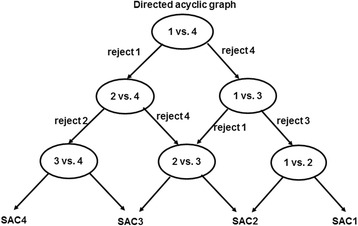
An example of directed acyclic graph (DAG) for identifying severity level (SAC1: extreme risk; SAC2: high risk; SAC3: medium risk; SAC4: low risk): each node is a binary classifier for two levels of incidents. A decision of rejecting one of the two levels is made at each node
4. Performance evaluation
  Our aim was to identify specific incident types, thus F-score, precision and recall measures were evaluated per type. We calculated the probability that a specific incident type or severity level was classified as such (e.g. % of falls correctly identified among the test set for falls). Performance in identifying incident types and severity levels was also examined using confusion matrices.
  Overall classification performance was examined using average measures, micro-averaging and macro-averaging, these are widely accepted and commonly used in many multiclass classification studies (Additional file 1: Appendix C) [41]. The macro-averaged measures of precision, recall and F-score are the simple average over all classes with equal weight to each incident type while micro-averaged measures are based on the cumulative number of true positives (tp), true negatives (tn), false positives (fp) and false negatives (fn) per type [41]. We used micro-averaged F-scores to select the best performing classifiers because this measure evaluates classification performance over the whole dataset.

## Results

### Overall classifier performance

Testing against the benchmark, original and independent datasets showed that OvsO ensembles of SVM RBF with binary count feature extraction were the most effective combination to identify incident type and severity level (Additional file 1: Appendix D). With OvsO, the DAG decision-making scheme performed slightly better than voting across each testing dataset. Using this most effective combination the overall performance of classifiers was then examined (Table 2). For incident type, the average F-score across all types was 78.3% on the testing datasets of benchmark and 73.9% on original but slightly worse on the independent testing dataset (68.5%). For severity level, the average F-score across all levels was 62.9% on the benchmark, 50.1% on the original and 52.7% on the independent datasets. Detailed results including performance with different combinations of binary classifier ensembles, feature extraction methods, and decision-making schemes are given in Additional file 1: Appendix D.

**Table 2:** Classifier performance (recall, precision and F-score). SVM RBF with binary count feature extraction was the most effective combination to identify incident type and severity level

	Benchmark			Original			Independent		
	Recall	Precision	F-score	Recall	Precision	F-score	Recall	Precision	F-score
Incident type ^a^	*78.3*	*78.3*	*78.3*	*73.9*	*73.9*	*73.9*	*68.5*	*68.5*	*68.5*
*Falls*	96.2	83.3	89.3	95.6	96.6	96.1	91.3	86.5	88.8
*Medications*	76.9	76.9	76.9	80.9	91.7	85.9	81.1	78.6	79.8
*Pressure injury*	88.5	100.0	93.9	89.2	86.8	88.0	96.8	76.0	85.2
*Aggression*	92.3	88.9	90.6	81.6	76.9	79.2	81.5	62.2	70.6
*Documentation*	46.2	63.2	53.3	46.2	31.6	37.5	47.6	16.0	24.0
*Blood products*	80.8	95.5	87.5	100.0	62.5	76.9	83.1	43.0	56.6
*Patient identification*	84.6	61.1	71.0	71.4	25.0	37.0	23.3	44.4	30.5
*Infection*	92.3	88.9	90.6	83.3	38.5	52.6	40.9	13.2	20.0
*Clinical handover*	80.8	65.6	72.4	71.4	18.5	29.4	37.9	14.3	20.8
*Deteriorating patient*	92.3	85.7	88.9	100.0	25.0	40.0	21.4	17.6	19.4
*Others*	30.8	50.0	38.1	54.7	85.3	66.7	57.1	87.0	69.0
SAC level ^a^	*62.9*	*62.9*	*62.9*	*50.1*	*50.1*	*50.1*	*52.7*	*52.7*	*52.7*
*SAC1*	82.8	92.3	87.3	84.0	11.2	19.8	82.6	6.8	12.5
*SAC2*	41.4	60.0	49.0	43.2	7.2	12.3	16.2	9.6	12.0
*SAC3*	44.8	54.2	49.1	35.9	52.3	42.6	46.9	49.8	48.3
*SAC4*	82.8	52.2	64.0	62.4	61.2	61.8	58.3	61.8	60.0

^a^Micro-averaging measures

### Identifying incident types

**Fig. 3:**
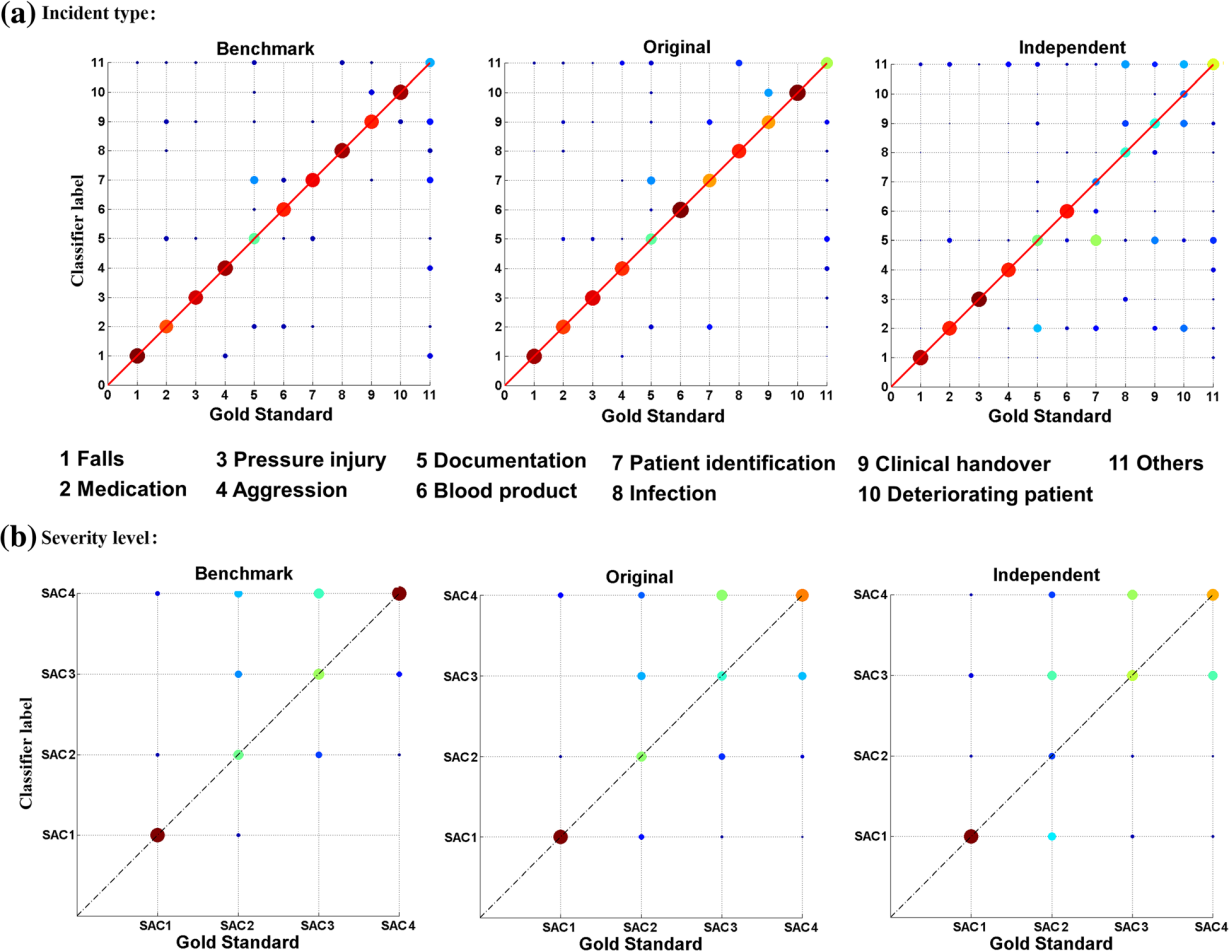
Confusion matrices for the best performing classifiers. Matrices were normalized by subset size; warmer colors indicate more correctly identified incidents (SAC1: extreme risk; SAC2: high risk; SAC3: medium risk; SAC4: low risk)

We found that classifiers using the most effective combination (i.e. OvsO ensembles of SVM RBF with binary count feature extraction) were robust in identifying four types of incidents including falls, medications, pressure injury, and aggression (Table 2). In the benchmark and original datasets, recall for incidents about blood products was comparable but marginally poorer with the independent dataset (individual precision 43%). For patient identification, infection, clinical handover and deteriorating patient, the classifiers achieved high F-scores on the benchmark dataset but performed poorly on the original and independent datasets. High recall along with low precision was achieved on the original dataset, but both precision and recall were poor on independent dataset. The classifiers performed relatively worse on identifying documentation reports, achieving an F-score of 24.0–53.3% across the testing datasets. Documentation was more likely to be misidentified as patient identification and medications (Fig. 3).

### Identifying severity levels

Testing on the benchmark dataset showed that the classifiers performed well in identifying SAC1 (F-score 87.3%) and SAC4 (F-score 64%; Table 2). With the original and independent datasets, high recall (82.8–84%) was achieved for SAC1 incidents but precision was poor (6.8-11.2%). For SAC3 and SAC4 performance was consistent across each testing dataset. However, identification of SAC1 and SAC2 in the original and independent datasets was poorer compared to the benchmark. F-scores for SAC1 decreased from 87.3 to 19.8% and 12.5% in the original and independent datasets.

## Disussion

### Main findings and implications

We evaluated text classification using binary classifier ensembles and our results demonstrate that this approach can identify reports about falls, medication, pressure injury and aggression as well as reports about extreme and low risk events. Classifiers were trained using balanced datasets from a state-wide incident reporting system and then evaluated on balanced and stratified subsets that were set aside for testing. We found that performance was comparable to a stratified dataset drawn from an independent hospital reporting system. This indicated generalizability of the approach showing that the classifiers for falls, medications, pressure injury, aggression, extreme and low risk events can be used in a real-world setting to collate and examine data from disparate incident reporting systems to support learning from patient safety incidents at regional, national and international levels. Even so, it should be emphasized that automated identification of incident reports is not intended as a replacement for expert review. Manual analysis provides insights that cannot be captured by any automated methods. However, when human resources are lacking, automated methods can reduce the effort spent in identifying common incident types, and provide small volumes of like incident reports for further investigation by experts [42]. Automated methods are only a first step in characterizing any cluster of incidents [43].

### Identification of common and rare classes

An important finding of this study is that SVM RBF with binary count identified the most common incident types across the three datasets including falls, medications, pressure injury and aggression. These types made up 54% of all reported incidents (Table 1). Similarly, performance was good for SAC4 incidents which made up over half of all reported incidents (52%; Table 1). In contrast, classifiers trained on balanced datasets tended to be weaker when identifying rarer types in stratified datasets such as patient identification, infection, clinical handover, and deteriorating patient, which made up 5-6% of all reported incidents (Table 1).

Performance for patient identification and clinical handover with balanced datasets was comparable with our previous study which examined binary classifiers using SVM RBF [23] (F-score_previous_ = 94.35 and 88.71% respectively vs. F-score_current_ = 71 and 72.4%). Similarly, for SAC1 incidents, the classifiers performed well on the balanced dataset (recall = 82.8%, precision = 92.3%) and effectively detected true positives when tested with stratified datasets (recall: original = 84%, independent = 82.6%). These results were also comparable with our previous study to identify extreme-risk events (recall = 83%, precision = 88%) [25].

One possible way to improve the identification of rare classes, especially SAC1 incidents, is to use rule-based methods [44] and active learning [45, 46] that involve expert knowledge and incorporate specific criteria for identifying incidents. An interim solution might be to review rare classes flagged by classifiers, which is practical because overall volumes in real-world datasets will be low. For example, 8 out of 444 reports in the original dataset were misidentified as infection. With around 132,861 reports in 2012, 46 false positives would need to be checked by experts per week. Similarly, poor precision was observed in identifying SAC1 in the stratified datasets alongside very promising recall measures. As SAC1 incidents are always subject to a thorough investigation such as root-cause analysis, any false positives can easily be detected by experts initially screening the incidents flagged by a classifier. Another way to improve identification of rare classes is to use balanced training sets by oversampling rare classes or down sampling common classes into multiple subsets and then build up an ensemble of binary classifiers between each subset of a common class and a rare one [47]. Sensitivity can also be improved by increasing misclassification costs for rare classes so that they gain more importance during classifier training [47].

We observed a drop in the average F-score when the classifiers were tested with stratified datasets (Table 2: incident type 9.8% and severity 10.2%). This is expected with supervised classification methods because the performance of classifiers trained on balanced datasets tends to degrade when models are applied to stratified datasets. Minor differences in terminology and linguistic styles may have also contributed to poorer performance on the independent dataset.

For overall classifier performance, the average recall and precision were identical. This was because the sum of individual (tp + fp)_i_ and (tp + fn)_i_ turned out to be the same as the total testing size even though the number of false negatives and false positives for each class were different.

Overall, lower performance was observed with severity levels compared to incident types. This is because each SAC level included multiple incident types making it harder to obtain distinct vocabularies between levels. For instance, SAC1 incidents involved falls, patient identification, clinical handover and others while falls, medications, pressure ulcer and patient identification were observed in each of the other levels (SAC2 to SAC4) [48]. The relationships between specific incident types and severity levels were not investigated, and would be worthwhile to study further. Overall, identification of SAC2 and SAC3 was worse than SAC4, with poor precision and recall in original and independent datasets (Fig. 3). In addition to the presence of multiple incident types in a severity level group, this may be a reflection of the inherent difficulties experienced by humans in using the SAC matrix where there is a significant overlap in the consequences for SAC2 and SAC3 [2]. Consequently, the outer classes, SAC1 and SAC4, tend to be identified more easily than SAC2 and SAC3 where boundaries are harder to distinguish.

### Identifying incidents with implicit causes and results

We observed that classifiers failed when causes and consequences of incidents were implicitly described in reports. This might be due to the bag-of-words model which does not account for text semantics. For instance, an incident about a deteriorating patient with a long list of medications and their doses in the narrative was misidentified as a medications problem (Additional file 1: Appendix E). Other instances were incidents misidentified as SAC1 where reports contained words that were associated with true positives (e.g. ‘death’, ‘suicide’, ‘high risk’, ‘police notified’, ‘incorrect patient’ and ‘infection’; Table 3) [49]. A third scenario involved false positive incidents with minor clinical consequence or near miss events where potential adverse outcomes had been avoided but were described in incident reports (70% of false positives in original and 85% in the independent dataset). For example, some reports described situations where there was a high risk for a fall with treatments involving neuro observation, vital signs checking, and CT brain scanning, or if a patient suffered extreme pain or hit his head. In other cases, patient identification incidents containing the phrase ‘incorrect patient’ were misidentified as SAC1 when no patient harm was reported. Similarly, reports about patients who had absconded were misidentified as SAC1 because they involved police notification. Our error analysis also uncovered 18 SAC1 incidents involving patient deaths that had been missed by human classifiers (e.g. a patient died in operation room, ambulance, ICU, or during a transfer; Table 3). In summary, these patterns reflect both the strengths and limitations of the bag-of-words model, suggesting that a combination approach that considers the meaning and order of words might be required. This should be the subject of further investigation using feature extraction methods such as UMLS semantic types and N-grams which have been shown to be effective in similar tasks like detection of adverse drug reactions [50].

**Table 3:** Key words associated with SAC1 incidents [50], along with excerpts from reports that were misidentified

Key words	Misidentified by machine classifiers (false positives)	Misidentified by humans (false negatives)
death	problem with death certificate, police notified	patient died in operation room, ambulance or ICU, or died when transferring
suicide	suicide or suspected suicide outside of hospital	inpatient suicide
high risk	high fall risk mentioned e.g. patient suffered extreme pain or hit their head, neurological observation, vital signs checked, CT scan of brain	
	high risk medication, drug overdose or wrong medicine	
police notified	absconded patients with mental health problems did not return from planned leave, police intervention	
incorrect patient	duplicate CT scans due to problem with patient identification	incorrect site for patient procedure
infection	patient had infection in hospital	more than two staff infected by patients
blood transfusion reaction		shortly after commencing the flebogamma infusion patient reacted to the medication with shortness of breath, chest tightness, vomiting and diarrhea
aggression		patient with mental health problems or Hepatitis C infection assaulted staff

### Multiclass nature of incident reports

We found that around 30% of reports could potentially be related to more than one incident type. This tended to be more pronounced for some incident types (patient identification: 62%; clinical handover: 42%; and deteriorating patient: 42%) and posed a challenge for our classifiers that were built to identify a single type. As shown in Table 2, classifiers performed well in identifying more distinct incident types, such as falls, medications, pressure injury and aggression incidents. However, documentation was frequently misidentified as this type always occurred alongside other incident types (Table 2). In both the AIMS and Riskman datasets, we found that documentation issues were reported alongside patient identification (47, 77%), clinical handover (39, 30%) and medications incidents (68, 75%) confirming that more than one incident type may be applicable in some cases. For instance, in one report, ‘label Z for patient X was placed incorrectly onto the specimen that belongs to patient Y’ showing that patient identification and documentation errors were involved (Additional file 1: Appendix E). One possible solution to this problem is to use multiple labels in training and testing using multi-label classifiers [51, 52]. Further work is required to investigate this approach and its potential to improve the performance of classifiers in a real-world setting.

### Limitations

There are several limitations. Firstly, we used datasets from one Australian state. Therefore our classifiers may not be generalizable to other jurisdictions and regions with different reporting, linguistic styles and terminology. Secondly, we exclusively evaluated logistic regression and SVM using balanced datasets to train classifiers. We did not examine the use of stratified datasets for classifier training because we were restricted to using incidents that had been reported over a 12-month period. Given the class imbalance between incident types a stratified training set may have worked better to identify rarer types. For testing, the AIMS dataset may have had too few instances to evaluate performance. This was a limitation of the number of incident reports that were made available to us for this study. For example, of the 350 reports about falls, which is the most common incident type, 260 were used for training and testing via cross-validation leaving only 90 for a separate test set. Thus the stratified AIMS dataset had few instances of the rarer incident types.

## Conclusion

The use of text-based binary classifier ensembles is a feasible approach for automatically identifying incidents by type and severity. OvsO ensembles of binary SVM RBF classifiers with binary count feature extraction was the most effective combination. Despite its limitations automated identification can provide a more efficient way to provide initial review of incident reports so that human resources can be redirected to detailed classification, and remedial actions can then be triggered more quickly to respond to emerging safety issues. In addition, automated identification can help to find misidentified incidents and enhance data quality. Testing against stratified AIMS and independent Riskman systems suggests that our method may be transferable to other incident reporting systems nationally and internationally. Given that reports often relate to more than one incident type, classifier performance could be improved using multiple labels for training and testing.

## Additional file

Additional file 1: Five appendix sections are included in this file. **Appendix A.** Examples of reports from the AIMS and Riskman systems. **Appendix B.** Definition of incident types used by experts to label reports. **Appendix C.** Classification performance measures. **Appendix D.** Classification performance. **Appendix E.** Examples of incident reports. (DOCX 57 kb)

## Abbreviations

AIMS: Advanced Incident Management System
OvsA: One-versus-all
OvsO: One-versus-one
RBF: Radial-basis function
SAC: Severity assessment codes
SVM: Support vector machine
