# Supplementary material for: Using multiclass classification to automate the identification of patient safety incident reports by type and severity
Source: BMC Med Inform Decis Mak. 2017 Jun 12;17:84. doi: 10.1186/s12911-017-0483-8 (PMC5468980; doi:10.1186/s12911-017-0483-8)
Supplement: Additional file 1: — Five appendix sections are included in this file. Appendix A. Examples of reports from the AIMS and Riskman systems. Appendix B. Definition of incident types used by experts to label reports. Appendix C. Classification performance measures. Appendix D. Classification performance. Appendix E. Examples of incident reports. (DOCX 57 kb) [file 12911_2017_483_MOESM1_ESM.docx]

**Appendix A: Examples of reports from the AIMS and Riskman systems**

Table A1: Example of an incident reported to the AIMS system

| **Element of report** | **Example** |
| --- | --- |
| 1. Incident ID | *1127813-20* |
| 2. Incident date | *9/26/2011* |
| 3. Incident time | *8:45 PM* |
| 4. Specific service | *Emergency medicine; Aged Care - Geriatrics* |
| 5. Incident type(s) | *Fall* |
| 6. Incident description | *Patient standing at the end of the bed and nursing staff had just taken observations. Nurse was about to check BSL and patient lowered herself to the floor. Patient did not hit her head. Dr was on ward at time and came to review. Patient appeared unresponsive yet did not allow her hand to hit her head when examined by Dr.* |
| 7. Outcome | *---* |
| 8. Actual SAC | *4* |
| 9. Action taken | *Dr informed. Observation BSL and neuro observation taken. Patient walked herself to chair.* |
| 10. How could the incident have been prevented? | *---* |
| 11. Results of incident | *After speaking with patient nursing staff and doctor it looks as if the patient is having pseudo falls making sure that when it happens someone is around and does not actually fall or injure herself. Patient has some significant mental health issues and has presented to hospital many times. On each occasion when it is near time to go home puts up obstacles.* |

Table A2: Example of an incident reported to the Riskman system

| **Element of report** | **Example** |
| --- | --- |
| 1. Incident ID | *104144* |
| 1. Incident date | *5/27/2010* |
| 1. Incident time | *09:45* |
| 1. Incident type(s) | *Fall* |
| 1. Incident description | *Ms. X fell down a step on level 6 of the Y building* |
| 1. Detail | *Ms. X was part of the choir who were singing at the opening ceremony of the Z Centre. The accident happened at approx. 0945 Ms. X was assisted by other staff who were present and was taken in a wheel chair to the ED by a nurse and a volunteer. She was obviously in a lot of pain.* |
| 1. Comments | *---* |
| 1. Outcome | *Moderate Injury, temporary impairment* |
| 1. Actual SAC | *3* |
| 1. Action taken | *---* |
| 1. Treatment | *The accident was witnessed by other members of the choir and staff in attendance for the Opening* |
| 1. Investigation findings | *Though the edge of the step was painted with a yellow stripe and there were signs warning of the step it was easy not to see it* |
| 1. Implemented controls | *The 6th floor of the Centre is still not complete and so it is to be expected /hoped that this hazard will not be there in the finished floor* |

**Appendix B: Definition of incident types used by experts to label reports**

Table B1: Criteria used by experts and examples of incident of each type

| **Incident type** | **Definition and examples** |
| --- | --- |
| **Fall** | Classifying reports by details related to a fall.  *For example, a patient fell from a theatre bed; a patient was lowered to the ground by a staff member after losing their balance; a patient was found on the floor and it could not be confirmed that a fall took place; a disorientated patient fell while ambulating after forgetting to use their walking frame.* |
| **Medication** | Medication or intravenous fluid incidents.  *For example, medication prescribing errors; incorrect intravenous fluid infusion rates.* |
| **Pressure injury** | Either new pressure ulcers or the worsening of pre-existing pressure ulcers.  *For example, a bed bound patient develops a pressure area despite all appropriate preventative measures being implemented; an existing sacral pressure area develops into a pressure sore.* |
| **Aggression** | Classifying reports by details of the aggressive person (or the perpetrator) or the victim of an aggressive incident.  *For example, a patient punching another person; a person making death threats.* |
| **Documentation** | An incident involving a problem with any written, typed, drawn, stamped or printed text/information and/or any document into which it has been entered.  *For example, a patient's medication chart is filed into another patient's medical records; a patient's surname is misspelled on their wrist ID band; an x-ray request form is used to request a blood test; a treatment order is ambiguous or difficult to read; an administered treatment is not signed for as required; incorrectly labelled specimens.* |
| **Blood product** | Blood or blood product transfusion processes, dispensing or quality problems.  *For example, a patient suffers an anaphylactic reaction to a blood transfusion; a blood unit is mislabeled during the dispensing process; blood is stored at the incorrect temperature; incorrect blood pack dispensed from transfusion service.* |
| **Patient identification** | Failure to correctly identify patients, and/or correlate their clinical information with an intended, or previous test or procedure.  *For example, the test or procedure is performed on the wrong patient; or the wrong procedure or test is performed; or the wrong side, or site, of the patient is used.* |
| **Infection** | Infections or infestation acquired as a result of hospitalisation.  *For example, a post-operative wound infection; septicaemia as a result of hospitalisation; an infected IV (intra-venous) cannula site.* |
| **Clinical handover**  **[1-3]*** | The transfer of professional responsibility and accountability for some or all aspects of care for a patient, or group of patients, to another person or professional group on a temporary or permanent basis.  *For example, a post-operative patient is transferred to the ward in an unstable situation with raised blood pressure; or a patient arrives in a ward from emergency department without handover.* |
| **Deteriorating patient**  **[4]*** | Failure to recognise and/or respond to clinical deterioration of patients, or failure in systems and processes to detect clinical deterioration and/or respond effectively to the deteriorating patient. The criteria for calling a MET (Medical Emergency Team) were used to classify a deteriorating patient:   1. Staff member is worried about the patient 2. Airway    1. Noisy breathing / stridor 3. Breathing    1. Acute change in respiratory rate to < 8 or > 30 breaths / min    2. Acute change in pulse oximetry saturation to < 90% despite oxygen administration 4. Circulation    1. Acute change in heart rate to < 40 or > 130 beats / min    2. Ischemic chest pain    3. Acute change in systolic blood pressure to < 90 mmHg    4. Acute change in urinary output to < 50 mL in 4 hrs 5. Conscious state   5.1 Acute change in conscious state  5.2 Multiple seizures  *For example, patient deteriorated over the evening shift with changed level of consciousness requiring clinical review.* |

*1. Australian Commission on Safety and Quality in Health Care. The OSSIE Guide to Clinical Handover Improvement. Sydney: ACSQHC; 2009.

1. Australian Medical Association. Safe Handover: Safe Patients. AMA; 2006.
2. Jorm C, Iedema R. Innovative Approaches to Enhancing Clinical Handover. Paris: International Forum on Quality and Safety in Health Care; 2008.
3. Jones D, Duke G, Green J, Briedis J, Bellomo R, Casamento A, Kattula A, Way M: Medical Emergency Team syndromes and an approach to their management. Crit Care 2006, 10:R30

Table B2: 10 ten incident types used to build the ‘Others’ type

| Behavior/human performance | Accident/occupational health and safety |
| --- | --- |
| Medical device/equipment/property | Organization management/service |
| Nutrition | Complaint |
| Pathology/laboratory | Security |
| Building/fittings/fixtures | Oxygen/gas/vapour |

**Appendix C: Classification performance measures**

We used both micro- and macro-averaging. Micro-averaging is based on the cumulative number of true positive (tp), true negative (tn), false positive (fp) and false negative (fn) from each class and then calculate a performance measure while macro-averaging is the simple average of individual measures (precision, recall and F-score) from each class [35]. (Table C1).

Table C1: Averaged precision, recall and F-score were used to evaluate classifier performance.

| **Measure** | **Formula** | **Evaluation focus** |
| --- | --- | --- |
| ***Precision_µ*_*** | $\frac{\sum_{i=1}^{l} {tp}_{i}}{\sum_{i=1}^{l} {tp}_{i}+{fp}_{i}}$ | Agreement of Gold Standard labels with those of a classifier if calculated from sums of per-class decisions |
| ***Recall_µ_*** | $\frac{\sum_{i=1}^{l} {tp}_{i}}{\sum_{i=1}^{l} {tp}_{i}+{fn}_{i}}$ | Effectiveness of a classifier to identify class labels if calculated from sums of per-class decisions. |
| ***F-score_µ_*** | $\frac{{(\beta}^{2}+1)Precision\mu Recall\mu}{\beta^{2}Precision\mu+Recall\mu}$ | Relations between data’s positive labels and those given by a classifier based on sums of per-class decisions. |
| ***Precision_M*_*** | $\frac{\sum_{i=1}^{l} \frac{{tp}_{i}}{{tp}_{i}+{fp}_{i}}}{l}$ | An average per-class agreement of the data class labels with binary classifier ensembles. |
| ***Recall_M_*** | $\frac{\sum_{i=1}^{l} \frac{{tp}_{i}}{{tp}_{i}+{fn}_{i}}}{l}$ | An average per-class effectiveness of a classifier to identify class labels. |
| ***F-score_M_*** | $\frac{{(\beta}^{2}+1){Precision}_{M}{Recall}_{M}}{\beta^{2}{Precision}_{M}+{Recall}_{M}}$ | Relations between data’s positive labels and those given by a classifier based on a per-class average. |

*µ**: micro-averaging measures

*_M_**: macro-averaging measures

*l* = number of classes

*tp_i_* = true positive, the number of incidents correctly classified to be in a given class *i*

*tn_i_ =* true negative, the number of incidents correctly classified not to be in a given class *i*

*fp_i_* = false positive, the number of incidents falsely classified to be in a given class *i*

*fn_i_* = false negative, the number of incidents falsely classified as not to be in a given class *i*

Precision = % of true positives detected in relation to the total number of incidents classified for a class

Recall = % of true positives detected in relation to the actual number of incidents in a class

F-score = harmonic mean of precision and recall, where *β* enables F-score to favor either precision or recall. Precision and recall are given equal weight by setting *β* to 1.

Table C2: Averaged classifier performance (micro and macro-averaged recall, precision and F-scores). SVM RBF with binary count feature extraction was the most effective combination to identify incident type and severity level.

|  | | **Micro averaging** | | | **Macro averaging** | | |
| --- | --- | --- | --- | --- | --- | --- | --- |
|  |  | Precision | Recall | F-score | Precision | Recall | F-score |
| **Incident type** | **Benchmark** | 78.3 | 78.3 | 78.3 | 78.1 | 78.3 | 78.2 |
|  | **Original** | 73.9 | 73.9 | 73.9 | 58.0 | 79.5 | 68.8 |
|  | **Independent** | 68.5 | 68.5 | 68.5 | 49.0 | 60.2 | 54.6 |
| **Severity level** | **Benchmark** | 62.9 | 62.9 | 62.9 | 64.7 | 62.9 | 63.8 |
|  | **Original** | 50.1 | 50.1 | 50.1 | 33.0 | 56.4 | 44.7 |
|  | **Independent** | 52.7 | 52.7 | 52.7 | 32.0 | 51.1 | 41.5 |

**Appendix D: Classification performance**

**Incident type**

Table D1: Micro-averaged F-scores with different classifier ensembles, feature extraction, and decision making strategies. The best performance in identifying incident type is highlighted in bold.

| Classifier ensemble | Base classifier | Feature extraction | benchmark | | original | | independent | |
| --- | --- | --- | --- | --- | --- | --- | --- | --- |
|  |  |  | **voting** | **DAG** | **voting** | **DAG** | **voting** | **DAG** |
| OvsO | **SVM RBF** | tf-idf | 73.4 | 74.1 | 66.4 | 67.8 | 60.3 | 61.8 |
|  |  | term frequency | 65.0 | 65.0 | 59.0 | 60.6 | 47.1 | 50.2 |
|  |  | **binary count** | 78.0 | **78.3** | 73.4 | **73.9** | 66.4 | **68.5** |
|  | SVM linear | tf-idf | 75.5 | 75.5 | 67.6 | 69.4 | 62.32 | 61.3 |
|  |  | term frequency | 69.6 | 69.9 | 66.7 | 68.0 | 58.8 | 59.7 |
|  |  | binary count | 76.9 | 76.9 | 71.9 | 72.5 | 61.6 | 64.8 |
|  | Logistic regression | tf-idf | 23.4 | 18.9 | 39.6 | 30.4 | 30.1 | 30.2 |
|  |  | term frequency | 11.5 | 10.1 | 25.9 | 30.2 | 29.1 | 31.6 |
|  |  | binary count | 14.3 | 14.7 | 30.0 | 32.2 | 45.9 | 47.6 |
| OvsA | SVM RBF | tf-idf | 72.7 | * | 64.9 | * | 60.3 | * |
|  |  | term frequency | 58.0 | * | 52.3 | * | 49.5 | * |
|  |  | binary count | 75.9 | * | 69.4 | * | 63.5 | * |
|  | SVM linear | tf-idf | 73.1 | * | 67.3 | * | 61.0 | * |
|  |  | term frequency | 65.7 | * | 63.1 | * | 57.2 | * |
|  |  | binary count | 74.8 | * | 68.7 | * | 59.3 | * |
|  | Logistic regression | tf-idf | 54.6 | * | 64.9 | * | 59.6 | * |
|  |  | term frequency | 16.8 | * | 9.5 | * | 9.2 | * |
|  |  | binary count | 37.4 | * | 56.1 | * | 60.4 | * |

*, results not available for OvsA ensembles with DAG.

**Severity level**

Table D2: Micro-averaged F-scores with different classifier ensembles, feature extraction, and decision-making strategies. The best performing classifier is highlighted in bold.

| Classifier ensemble | Base classifier | Feature extraction | benchmark | | original | | independent | |
| --- | --- | --- | --- | --- | --- | --- | --- | --- |
|  |  |  | **voting** | **DAG** | **voting** | **DAG** | **voting** | **DAG** |
| OvsO | **SVM RBF** | tf-idf | 62.07 | 59.48 | 34.75 | 37.52 | 32.69 | 35.58 |
|  |  | term frequency | 51.72 | 50.00 | 34.71 | 39.34 | 38.17 | 42.64 |
|  |  | **binary count** | 62.07 | **62.93** | 47.34 | **50.11** | 49.66 | **52.67** |
|  | SVM linear | tf-idf | 56.90 | 56.90 | 35.81 | 37.34 | 34.24 | 36.74 |
|  |  | term frequency | 57.76 | 54.31 | 35.78 | 40.00 | 27.90 | 33.90 |
|  |  | binary count | 62.07 | 58.62 | 40.60 | 43.27 | 34.49 | 38.72 |
|  | Logistic regression | tf-idf | 43.9 | 44.83 | 37.15 | 39.21 | 39.39 | 44.25 |
|  |  | term frequency | 35.62 | 34.48 | 42.55 | 43.65 | 43.87 | 49.68 |
|  |  | binary count | 32.55 | 32.76 | 55.57 | 56.82 | 56.54 | 57.41 |
| OvsA | SVM RBF | tf-idf | 52.29 | * | 39.76 | * | 40.94 | * |
|  |  | term frequency | 45.69 | * | 38.68 | * | 40.34 | * |
|  |  | binary count | 59.48 | * | 44.80 | * | 49.97 | * |
|  | SVM linear | tf-idf | 52.59 | * | 38.64 | * | 37.71 | * |
|  |  | term frequency | 51.72 | * | 37.59 | * | 30.29 | * |
|  |  | binary count | 54.31 | * | 40.50 | * | 40.20 | * |
|  | Logistic Model | tf-idf | 50.00 | * | 40.10 | * | 37.17 | * |
|  |  | term frequency | 35.34 | * | 31.77 | * | 29.92 | * |
|  |  | binary count | 50.00 | * | 35.42 | * | 34.11 | * |

**Confusion matrices:**

Table D3: Confusion matrices for the best performing classifiers identifying incident types

| Classifier labels  Gold standard | *Falls* | *Medications* | *Pressure*  *injury* | *Aggression* | *Documentation* | *Blood*  *products* | *Patient*  *identification* | *Infection* | *Clinical*  *handover* | *Deteriorating patient* | *Others* |
| --- | --- | --- | --- | --- | --- | --- | --- | --- | --- | --- | --- |
| **Benchmark** | | | | | | | | | | | |
| *Falls* | 25 | 0 | 0 | 0 | 0 | 0 | 0 | 0 | 0 | 0 | 1 |
| *Medications* | 0 | 20 | 0 | 0 | 2 | 0 | 0 | 1 | 2 | 0 | 1 |
| *Pressure injury* | 0 | 0 | 23 | 0 | 1 | 0 | 0 | 0 | 1 | 0 | 1 |
| *Aggression* | 2 | 0 | 0 | 24 | 0 | 0 | 0 | 0 | 0 | 0 | 0 |
| *Documentation* | 0 | 2 | 0 | 0 | 12 | 1 | 7 | 0 | 1 | 1 | 2 |
| *Blood products* | 0 | 2 | 0 | 0 | 1 | 21 | 2 | 0 | 0 | 0 | 0 |
| *Patient identification* | 0 | 1 | 0 | 0 | 2 | 0 | 22 | 0 | 1 | 0 | 0 |
| *Infection* | 0 | 0 | 0 | 0 | 0 | 0 | 0 | 24 | 0 | 0 | 2 |
| *Clinical handover* | 0 | 0 | 0 | 0 | 0 | 0 | 1 | 0 | 21 | 3 | 1 |
| *Deteriorating patient* | 0 | 0 | 0 | 0 | 0 | 0 | 0 | 0 | 2 | 24 | 0 |
| *Others* | 3 | 1 | 0 | 3 | 1 | 0 | 4 | 2 | 4 | 0 | 8 |
| **Original** | | | | | | | | | | | |
| *Falls* | 86 | 0 | 0 | 0 | 0 | 0 | 0 | 1 | 0 | 0 | 3 |
| *Medications* | 0 | 55 | 0 | 0 | 5 | 0 | 0 | 2 | 4 | 0 | 2 |
| *Pressure injury* | 0 | 0 | 33 | 0 | 2 | 0 | 0 | 0 | 1 | 0 | 1 |
| *Aggression* | 2 | 0 | 0 | 40 | 1 | 0 | 1 | 0 | 0 | 0 | 5 |
| *Documentation* | 0 | 2 | 0 | 0 | 12 | 1 | 7 | 0 | 1 | 1 | 2 |
| *Blood products* | 0 | 0 | 0 | 0 | 0 | 5 | 0 | 0 | 0 | 0 | 0 |
| *Patient identification* | 0 | 1 | 0 | 0 | 0 | 0 | 5 | 0 | 1 | 0 | 0 |
| *Infection* | 0 | 0 | 0 | 0 | 0 | 0 | 0 | 5 | 0 | 0 | 1 |
| *Clinical handover* | 0 | 0 | 0 | 0 | 0 | 0 | 0 | 0 | 5 | 2 | 0 |
| *Deteriorating patient* | 0 | 0 | 0 | 0 | 0 | 0 | 0 | 0 | 0 | 1 | 0 |
| *Others* | 1 | 2 | 5 | 12 | 18 | 2 | 7 | 5 | 15 | 0 | 81 |
| **Independent** | | | | | | | | | | | |
| *Falls* | 796 | 4 | 1 | 8 | 8 | 0 | 0 | 4 | 1 | 2 | 48 |
| *Medications* | 0 | 854 | 1 | 3 | 83 | 10 | 1 | 3 | 11 | 2 | 85 |
| *Pressure injury* | 1 | 0 | 184 | 0 | 1 | 0 | 0 | 0 | 0 | 0 | 4 |
| *Aggression* | 5 | 8 | 1 | 397 | 10 | 0 | 0 | 3 | 5 | 0 | 58 |
| *Documentation* | 1 | 74 | 1 | 2 | 120 | 5 | 10 | 1 | 14 | 1 | 23 |
| *Blood products* | 0 | 3 | 0 | 0 | 4 | 49 | 0 | 1 | 0 | 0 | 2 |
| *Patient identification* | 0 | 10 | 0 | 0 | 44 | 8 | 20 | 1 | 0 | 0 | 3 |
| *Infection* | 0 | 0 | 2 | 0 | 1 | 0 | 0 | 9 | 4 | 0 | 6 |
| *Clinical handover* | 0 | 9 | 1 | 0 | 20 | 2 | 1 | 7 | 33 | 2 | 12 |
| *Deteriorating patient* | 0 | 3 | 0 | 0 | 1 | 0 | 0 | 0 | 3 | 3 | 4 |
| *Others* | 117 | 122 | 51 | 228 | 458 | 40 | 13 | 39 | 159 | 7 | 1644 |

**T**able D4: Confusion matrices for the best performing classifiers identifying severity levels

| **Classifier label**  **Gold standard** | SAC1 | SAC2 | SAC 3 | SAC 4 |
| --- | --- | --- | --- | --- |
| **Benchmark** | | | | |
| SAC 1 | 24 | 2 | 0 | 3 |
| SAC 2 | 2 | 12 | 7 | 8 |
| SAC 3 | 0 | 5 | 13 | 11 |
| SAC 4 | 0 | 1 | 4 | 24 |
| **Original** | | | | |
| SAC 1 | 21 | 1 | 0 | 3 |
| SAC 2 | 12 | 41 | 24 | 18 |
| SAC 3 | 97 | 336 | 789 | 976 |
| SAC 4 | 57 | 192 | 697 | 1573 |
| **Independent** | | | | |
| SAC 1 | 19 | 1 | 2 | 1 |
| SAC 2 | 32 | 17 | 39 | 17 |
| SAC 3 | 130 | 113 | 1224 | 1142 |
| SAC 4 | 100 | 47 | 1192 | 1874 |

**Appendix E: Examples of incident reports**

| **Reporting System** | **Gold standard** | **Secondary label** | **Classifier label** | **Narrative** |
| --- | --- | --- | --- | --- |
| **Reports with multiple incident types** | | | | |
| AIMS | documentation | others, pathology | patient ID | Episode label X for patient X was placed incorrectly onto the specimen that belongs to patient Y. |
| Riskman | documentation | blood product | clinical handover | Patient transferred from ED to CT room for nephrostomy. Patient not cannulated, nurse unable to give full handover as not been caring for patient and was only transferring patient. FFP not commenced as directed by Dr in MID. Nil ID band in situ. Cannula not patent for FFP. MID doctor consented patient, re-cannulated patient and FFP commenced as per policy. Requesting team contacted and asked to attend. ID band given to patient. Reassurance given, nephrostomy performed. |
| Riskman | patient identification | documentation | medication | Date of birth was incorrect on bradma, 2-12-1918 but patient states it is the 6-12-1918. Seretide dose was not written on the chart. Prednisolone was charted but was advised by team that it had been ceased. |
| **Reports with implicit mention of incident cause** | | | | |
| AIMS | infection | n/a | others | Spotless cleaner was about to attend to a VRE clean in CT room when Dr X dismissed cleaner from his duties and proceeded to put a patient from Emergency on uncleaned CT table. |
| Riskman | infection | n/a | pressure injury | Necrotic toes dressing changed, area cleansed with water and non-adhesive dressing applied, pain relief given. |
| Riskman | deteriorating patient | Others:complaint | medication | Patient admitted to Hospital 15/05/12. Patient had been charted Antihypertensives, Telmirsartan 40mg 0800hrs and Atenolol 50mg 2000hrs daily on admission. Antihypertensives was reported by son to have been ceased by GP recently. GP office had been contacted regarding current medication list, and wrong list had been faxed according to son. Lulworth Nursing Home had sent current medication list with patient on admission. Atenolol 50mg was given at 1950hrs on 16/05/12, BP125/80, and 1936hrs on 17/05/12, BP 109/66, then ceased on 18/05/12 at 1640hrs by medical team. Telmirsartan 40mg was given 0917hrs on 17/05/12, BP 101/50, then ceased on 17/05/12 at 1234hrs. Patient was paced on 18/05 for low BP 70/36. Also paced on 19/05 for low O2. Son been requesting to speak with team several times. Patient reported to have right hemiparesis which is new since admission. Pt has had a cerebral event which is new event, but not caused by low BP, or medication. Son concerned information is being withheld or covered up. Reassurance given. Son asking patient to be reviewed by an outside neurologist. Policy explained by Sr X and Dr Y and can be arranged through the correct channels. Son aware of contact person/fax number and information required. Son also asking for photo copy of notes/Obs chart. Son given contact number in executive and is aware a written request must be made. A/NUM has spoken to Rns involved & offered counselling if needed. NUM also spoke at length to a RN involved, offered access counselling & counselled RN. |
